# Ripples Have Distinct Spectral Properties and Phase-Amplitude Coupling With Slow Waves, but Indistinct Unit Firing, in Human Epileptogenic Hippocampus

**DOI:** 10.3389/fneur.2020.00174

**Published:** 2020-03-24

**Authors:** Shennan A. Weiss, Inkyung Song, Mei Leng, Tomás Pastore, Diego Slezak, Zachary Waldman, Iren Orosz, Richard Gorniak, Mustafa Donmez, Ashwini Sharan, Chengyuan Wu, Itzhak Fried, Michael R. Sperling, Anatol Bragin, Jerome Engel, Yuval Nir, Richard Staba

**Affiliations:** ^1^Department of Neurology and Neuroscience, Thomas Jefferson University, Philadelphia, PA, United States; ^2^Department of Medicine, Statistics Core, David Geffen School of Medicine at UCLA, Los Angeles, CA, United States; ^3^Department of Computer Science, University of Buenos Aires, Buenos Aires, Argentina; ^4^Department of Neurology, David Geffen School of Medicine at UCLA, Los Angeles, CA, United States; ^5^Department of Neuroradiology, Thomas Jefferson University, Philadelphia, PA, United States; ^6^Department of Neurosurgery, Thomas Jefferson University, Philadelphia, PA, United States; ^7^Department of Neurosurgery, David Geffen School of Medicine at UCLA, Los Angeles, CA, United States; ^8^Department of Neurobiology, David Geffen School of Medicine at UCLA, Los Angeles, CA, United States; ^9^Department of Psychiatry and Biobehavioral Sciences, David Geffen School of Medicine at UCLA, Los Angeles, CA, United States; ^10^Brain Research Institute, David Geffen School of Medicine at UCLA, Los Angeles, CA, United States; ^11^Department of Physiology and Pharmacology, Sackler School of Medicine and Sagol School of Neuroscience, Tel Aviv University, Tel Aviv-Yafo, Israel

**Keywords:** sleep, high-frequency oscillation, slow wave, epilepsy, hippocampus, ripple, fast ripple

## Abstract

Ripple oscillations (80–200 Hz) in the normal hippocampus are involved in memory consolidation during rest and sleep. In the epileptic brain, increased ripple and fast ripple (200–600 Hz) rates serve as a biomarker of epileptogenic brain. We report that both ripples and fast ripples exhibit a preferred phase angle of coupling with the trough-peak (or On-Off) state transition of the sleep slow wave in the hippocampal seizure onset zone (SOZ). Ripples on slow waves in the hippocampal SOZ also had a lower power, greater spectral frequency, and shorter duration than those in the non-SOZ. Slow waves in the mesial temporal lobe modulated the baseline firing rate of excitatory neurons, but did not significantly influence the increased firing rate associated with ripples. In summary, pathological ripples and fast ripples occur preferentially during the On-Off state transition of the slow wave in the epileptogenic hippocampus, and ripples do not require the increased recruitment of excitatory neurons.

## Introduction

**Figure 1:**
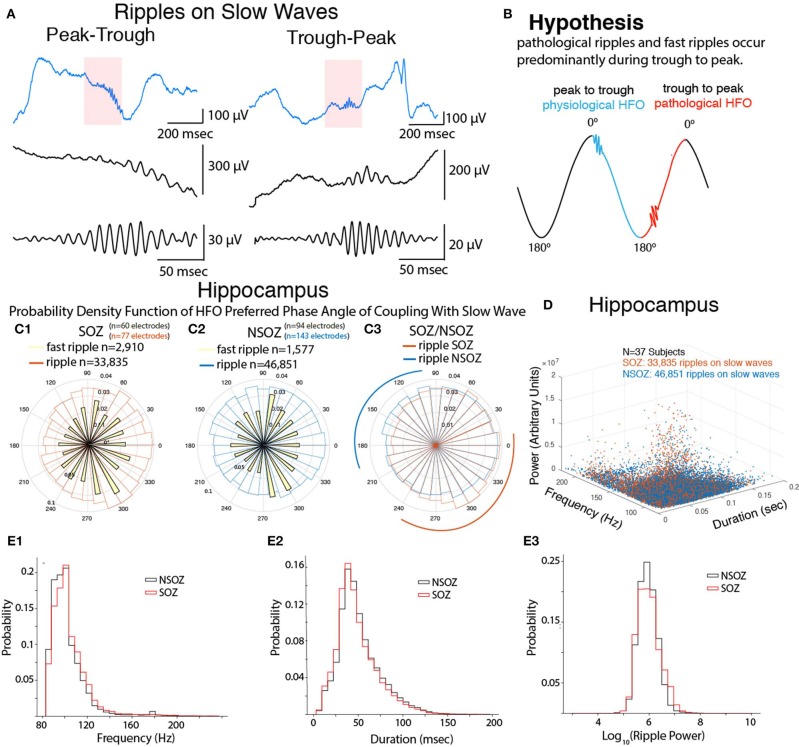
Fast ripples and ripples in the hippocampal seizure-onset zone (SOZ) are coupled with the trough-peak of the slow wave and exhibit distinct properties. **(A)** Example of ripples on slow waves during the peak-trough (or Off-On; left) and trough-peak (or On-Off; right) transition, (middle) ripples at an expanded time scale, (bottom) band-pass filtered ripples. **(B)** Illustration of main hypothesis pathological ripples and fast ripples preferentially occur during the trough-peak transition of the slow wave. **(C)** Normalized circular histogram [i.e., probability density function(PDF)] of fast ripple (yellow) and ripple [SOZ: red, non-SOZ(NSOZ): blue] preferred phase angle of coupling with respect to the slow wave measured in the SOZ **(C1)** and NSOZ **(C2)**. A direct comparison of ripple preferred phase angle of coupling in the SOZ and NSOZ is shown in **(C3)** where arcs represent regions where the SOZ PDF exceed the NSOZ PDF (red) and vice versa (blue). **(D)** Three dimensional scatter plot of ripple on slow wave properties in the SOZ (red) and NSOZ (blue). **(E)** Normalized histogram of the ripple on slow wave properties in the SOZ (red) and NSOZ (black).

In the epileptic brain, ripple oscillations (80–200 Hz) exhibit increased rates in epileptogenic mesial-temporal regions (1, 2). In the normal brain, ripples are important in memory consolidation during rest and sleep (3). Neocortical ripples during the trough-peak (or On-Off) state transition of the non-rapid eye movement (NREM) sleep slow wave are found at a higher density in epileptogenic tissue and are considered pathological (4–6) (Figures 1A,B). In the epileptogenic mesial temporal lobe, however, it is not clear if specific phases of the slow wave are associated with the generation of pathological ripples or fast ripples (200–600 Hz) (7, 8).

In the normal rat hippocampus CA1, ripples superimpose on sharp waves (<3 mV, 30–150 ms duration), which have the largest negative polarity in stratum radiatum and positive polarity in stratum pyramidale and oriens (9). Ripples are associated with a 5- to 6-fold increase in stratum pyramidale principal cell firing and 2- to 3-fold increase in stratum pyramidale and oriens non-principal firing. Both cell types discharge during the ripple trough, but non-principal cell firing is shifted a half-cycle with respect to principal cell discharges (10–12). Normal ripples are involved with memory consolidation and generated preferentially during the Off-On transition of the neocortical slow wave (13).

In the rat epileptogenic hippocampus, ripples can superimpose on interictal spikes (>4 mV, <30 ms) or on interictal spikes that have a positive polarity in stratum radiatum and negative polarity in stratum pyramidale and oriens (9, 14). Pathological ripples represent summated principal cell discharges with reduced non-principal firing (15–17) and could occur during On-Off transition of the slow wave.

Separating normal and pathological ripples in clinical studies, as in rat studies, would require electrodes with high spatial resolution, unit recordings, and precise anatomical localization of recording sites (18). This is not possible with clinical intracranial EEG (iEEG) electrodes, but these electrodes can record ripples during sleep slow waves and, combined with microelectrode recordings, could identify differences in EEG and unit firing that help to separate normal and pathological ripples. In the current study, we hypothesized that in the human epileptogenic hippocampus (i.e., seizure onset zone or SOZ), pathological ripples are generated during a preferred phase of the NREM sleep slow wave and involve a different level of principal cell firing than hippocampal ripples outside the SOZ. To evaluate this hypothesis, we analyzed iEEG and single unit recordings from the mesial temporal lobe of patients with drug-resistant focal epilepsy during NREM sleep.

## Methods

iEEG recordings that contained large amplitude slow wave activity associated with NREM sleep were retrospectively collected from 37 patients with mesial temporal or neocortical focal epilepsy. All patients underwent intracranial monitoring with depth electrodes between 2014 and 2018 at the University of California Los Angeles (UCLA) and Thomas Jefferson University (TJU) for the purpose of localization of the SOZ (Supplementary Table 1). The inclusion criteria for this patient cohort were a minimum of 4 h of interictal EEG recorded overnight that contained NREM sleep lasting between 10 and 60 min, sampled at 1 or 2 kHz, and was relatively free of muscle artifact. The 4-h recording criterion was used to exclude pre-ictal, ictal, and post-ictal episodes and to ensure sufficient epochs of slow wave sleep.

A second patient cohort included iEEG and single unit recordings from Behnke-Fried hybrid macro-micro electrodes obtained from five patients with focal epilepsy at UCLA who were monitored between 2007 and 2010 (19). In this second cohort, each of the macroelectrodes contained eight 40 μm platinum-iridium microwires that were designed to extend 3–5 mm beyond the distal tip and record extracellular wide bandwidth (1–6,000 Hz), local field potentials (LFP), and neuronal spikes (Supplementary Table 2). Both cohorts consisted of patients with mesial-temporal lobe and neocortical epilepsy who had similar medical histories and clinical features. This retrospective study was approved by the UCLA and TJU institutional review boards. All patients gave informed consent prior to participating in this research.

### Data Acquisition

The UCLA recordings were referenced to scalp electrode Cz, and the TJU recordings were referenced to an iEEG electrode positioned in the white matter per clinical protocol. Local field potential recordings were referenced locally to a ninth non-insulated microwire and synchronized with the iEEG recordings using a TTL pulse (19). For these sleep study recordings the iEEG recordings were synchronized with EOG and EMG recordings and the iEEG signals were referenced to earlobe electrodes for accurate comparison with scalp recordings (19). These recordings were part of a prior, larger study that included analysis of neocortical slow waves (19). NREM sleep was characterized by the predominance of irregular, large amplitude EEG activity comprised of slow waves, K-complexes, and spindles. Clinical iEEG sleep recordings at both UCLA and TJU (0.016–600 Hz) were acquired from 7 to 16 contact depth electrodes using a Nihon-Kohden 256-channel JE-120 long-term monitoring system (Nihon-Kohden America, Foothill Ranch, CA, USA) for patient cohort one, and a stellate EEG amplifier (XLTEK, San Diego, CA, USA) for patient cohort two. LFP recordings were acquired using a Neuralynx Cheetah (Neualynx, Bozeman, MT, USA) at a sampling rate of 28/30 kHz and bandpass-filtered between 1 and 6,000 Hz (19).

### Neuroimaging

The positions of surface and depth electrode contacts were obtained for all subjects from post-implantation computed tomography (CT) scans. Pre-implantation volumetric T1-weighted magnetic resonance imaging (MRI) scans were co-registered to the CT scans as well as to the Montreal Neurological Institute 152 (MNI152) standard brain to enable comparison of recording sites in a common space across subjects. Anatomic locations of the recording sites were derived by converting MNI coordinates to Talairach coordinates and querying the Talairach daemon. The SOZ was defined by visual inspection of ictal iEEG by clinicians at each of the data collection sites.

### Slow Wave-HFO Detection and Quantification

All iEEG recordings were imported from EDF format into Matlab v2017b (Natick, MA, USA). Subsequent processing steps for those recordings from macroelectrodes deemed suitable on the basis of visual inspection using Micromed™ Brainquick™ (Veneto TV, Italy) were performed using custom software developed in Matlab. The custom software generated HFO and EEG spike annotations in Brainquick™ that could be used to visually validate the accuracy of the detector (20).

In brief, the HFO detector reduced muscle and electrode artifacts in the iEEG recordings using a custom independent component analysis (ICA)-based algorithm (21). After applying this ICA-based method, ripples were detected in the referential and bipolar montage iEEG recordings per contact by utilizing a Hilbert detector, in which (i) a 1,000th order symmetric finite impulse response (FIR) band-pass filter (80–600 Hz) was applied, and (ii) a Hilbert transform was applied to calculate the instantaneous amplitude of this time series according to the analytic signal z(t), described in Weiss et al. (20) and Shimamoto et al. (21).

(1) $$\begin{array}{l} z\left(t\right)=a\left(t\right)e^{\wedge }i\phi \left(t\right) \end{array}$$

where a(t) is the instantaneous amplitude and ø(t) is the instantaneous phase of z(t). Following the Hilbert transform, (iii) the instantaneous HFO amplitude function [a(t)] was smoothed using moving window averaging, (iv) the smoothed instantaneous HFO amplitude function was normalized using the mean and standard deviation of the time series, and (v) a custom statistical threshold defined by the skewness of the normalized time series was used to detect the onset and offset of discrete/potential events.

HFO-like events can arise due to Gibb's phenomenon, i.e., high-pass filtering sharp transients, including epileptiform spikes. To distinguish authentic HFO during slow waves from authentic HFO on EEG spikes or spurious HFO due to filter ringing, we used a custom algorithm that performed topographic analysis of time-frequency plots for each HFO (22). The algorithm also measured the power, spectral content, and duration of each HFO. Both true HFO on EEG spikes and spurious HFOs were discarded from further analysis.

We identified ripple on slow wave (RoSW) events using the following approach. We first applied an optimized Hamming-windowed FIR band-pass filter between 0.1 and 2 Hz (eegfiltnew.m; EEGLAB, https://sccn.ucsd.edu/eeglab) to all the iEEG recordings optimally reducing phase distortion (6, 23). We then calculated the normalized instantaneous amplitude of the Hilbert transformed band-pass filtered signal (Equation 1). We used independent onset and offset normalized minimum amplitude (z-score) and duration criteria defined on the basis of visual inspection of the algorithm results to identify epochs in which slow oscillatory epochs appeared (6). After identifying the slow epochs, the corresponding epoch time stamps were used to classify the RoSW.

### Calculation of Ripple Phasors During Sleep Slow Wave

To assess phase-amplitude coupling we transformed each HFO into a HFO phasor (6), as described in Equation (2).

(2) $$\begin{array}{l} ve^{i\theta }=\overset{T}{\underset{t}{\sum }}a\left(t\right)e^{i\phi \left(t\right)} \end{array}$$

where *v* is the vector strength of the phasor, theta its preferred slow-wave phase angle, and *a*(*t*) and ø(*t*) the respective instantaneous HFO amplitude iEEG slow wave phase during the ripple across its duration [*t*:*T*], where *t* is the onset of the HFO and *T* is the offset. Thus, the preferred phase angle represents the mean phase angle of coupling between the ripple and slow wave.

### Single Unit Analysis

Extracellular action potentials were detected by high-pass filtering the microelectrode recordings above 300 Hz and applying a threshold at 5 SD above the median noise level (19). Detected events were categorized as noise, single-, or multi-unit activity using superparamagnetic clustering for unsupervised classification of each spike waveform (19). The stability of unit firing throughout the recording was assessed by inspecting the spike waveforms and inter-spike interval histograms. An inter-spike interval histogram with a clear refractory period of 2 ms or greater was considered a putative single unit; otherwise it was considered multiunit activity.

For each single unit mean action potential waveform we measured the peak amplitude asymmetry, a measurement of the relative differences in the peaks prior to and following the depolarizing spikes, the duration between the trough and the following peak, and half-width duration at half amplitude of the action potential waveform (19). We quantified single unit firing before, during, and after RoSW to generate binary vectors of the action potentials in 1-ms bins. For a 10 min episode of NREM sleep we computed the instantaneous phase value of the slow wave activity with respect to each action potential, and then repeated this analysis after removing action potentials associated with a ripple, i.e., action potentials within 250 ms a ripple. This interval surrounding the ripple was based on an analysis that found 47 ± 28% of all ripples occurred with an inter-ripple interval of <500 ms. Spike trains were smoothed with a 100 ms Gaussian kernel and then down-sampled to 100 Hz for comparison with ripples. A long-duration kernel was used because of the relatively sparse unit firing.

### Statistical Analysis

We used a non-linear, logistic mixed effects model to derive the probability for predicting the SOZ using the sin and cos of the slow-ripple phase angle and controlling for the following: duration, spectral frequency, and power of the ripple; and patient's gender, race, seizure type, seizure location, montage, reference electrode, risk factor, and MRI and PET findings. We used a log transformation on variables with non-normal distributions. Measures were clustered by patient by the random effect model. The analysis was stratified by anatomical location. *P*-values < 0.05 were considered statistically significant. All analyses will be performed using SAS v. 9.4 (SAS Institute Inc., Cary, NC, USA).

A linear, mixed effects model in SAS v. 9.4 was used to analyze changes in firing rate during the slow-ripple events (ripple peak amplitude ±25 ms) with respect to baseline firing. Two sets of analyses were performed. The first considered trials clustered by unit then by patient using unit-within-patient nested random effects. In the second, units were clustered by electrode using electrode-within-patient nested random effects. The models controlled for location, spectral frequency and power of the ripples, slow wave-ripple coupling as defined by slow wave peak-trough or trough-peak distribution, and location within the SOZ (i.e., etiology). Similar models were used to test the baseline firing rate and the overall firing rate.

## Results

We analyzed iEEG recordings during NREM sleep episodes from 37 patients with medically refractory epilepsy and electrodes implanted in the hippocampal gray matter (Supplementary Table 1). To examine if the phase of the slow wave correlates with the generation of fast ripples and ripples (Figures 1A,B) first we compared the probability density function (PDF) of the phase angles of coupling between fast ripples and slow waves (Figure 1C1), and the PDF of ripples and slow waves in the seizure onset zone (SOZ, Figure 1C1). Fast ripples were phase locked with the slow wave (Rayleigh's test, *Z* = 33.4, *p* < 1e-9) at a mean phase angle of 334 ± 121° and a maxima of 310° [trough-peak]. Ripples also exhibited strong phase locking with the slow wave (*Z* = 418, *p* < 1e-9) at a mean phase angle of 356 ± 121° and a maxima at 310° (Figure 1C1). By comparison, fast ripples in the NSOZ were not nearly as strongly phase locked as those in the SOZ (Figure 1C2, Rayleigh's test, *Z* = 9.16, *p* = 1e-4). The mean phase angle for fast ripples in the NSOZ was 19 ± 130° and the maxima was 290° (Figure 1C2). Ripples in the NSOZ were strongly phase locked to the slow wave but not as strong as in the SOZ (*Z* = 176, *p* < 1e-9), and at a different mean phase angle of 42 ± 140° and maxima of 10° (Figure 1C2).

Next, we compared ripples in the SOZ to those in the NSOZ (Figure 1C3). The PDF for ripples in the SOZ indicated ripples were more likely to occur between 240 and 10° [trough-peak] of the slow wave, whereas in the NSOZ ripples were more likely to occur between 90 and 200° [peak-trough] of the slow wave (Figure 1C3). A comparison of the ripple phase angles in the SOZ and NSOZ using both circular statistical methods (Kuiper's *p* < 0.001) and a logistic regression model (LRM, *p* < 0.0001) confirmed that the phase angles for ripple-slow wave coupling in the SOZ and NSOZ were distinct. The remaining analyses focused on ripples since, unlike fast ripples, they support physiological functions such as memory consolidation during sleep in the hippocampus, and our objective was to distinguish physiological from pathological ripples.

**Figure 2:**
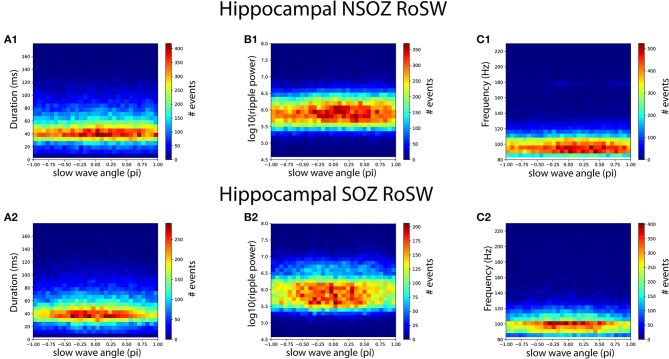
Longer duration, higher power, lower spectral content ripples on slow waves (RoSW) during the peak-trough transition were more frequent in the hippocampal non-seizure onset zone (NSOZ) than the SOZ. Histograms quantifying the number of RoSW events binned by **(A1,A2)** duration, **(B1,B2)** spectral power, and **(C1,C2)** spectral frequency and by the phase angle of coupling recorded from the NSOZ (top row) and the SOZ (bottom row).

We hypothesized that ripples on slow waves (RoSW) in the SOZ would have different spectral frequency, power, and duration than those in the NSOZ because pathological ripples with distinct properties should be over expressed in the SOZ. Analysis of these properties revealed an overlap of values between RoSW in the SOZ and NSOZ (Figures 1D,E). In spite of the overlap, however, the LRM found RoSW in the hippocampus SOZ had a higher spectral frequency (Figure 1E1, *p* < 0.001), shorter duration (Figure 1E2, *p* < 0.005), and lower power (Figure 1E3, *p* < 0.005) than those recorded in the NSOZ. As predicted by the LRM, there were more RoSW between 90 and 200° (peak-trough) transition with lower spectral content (Figure 2C), a longer duration (Figure 2A), and greater power (Figure 2B) in the NSOZ than in the SOZ (Figure 2). Other factors in the LRM, such as recording montage (referential or bipolar), electrode reference, and clinical metadata, did not affect these results.

Evidence suggests slow waves modulate unit firing and, in the hippocampus, RoSW could have a stronger effect on unit firing both in the SOZ and, possibly, remote brain areas. To evaluate unit firing modulation, we analyzed slow waves and ripples recorded from the most distal contact on the macroelectrode and single unit firing from the adjacent microelectrode during NREM sleep from five patients with medically refractory epilepsy (Supplementary Table 2). We isolated 59 (39 in SOZ and 20 in NSOZ) putative excitatory and one inhibitory single unit on the basis of waveform morphology and firing rate characteristics from 430 min of sleep recorded in these 5 patients from hippocampal and extra-hippocampal structures.

First we analyzed unit firing modulation during all slow wave activity and then repeated the analysis after removing action potentials associated with ripples (see section Methods). For the 39 neurons in the SOZ, unit firing was strongly modulated by the slow wave (*Z* = 45.4, *p* < 1e-9) and the highest firing probability was at a mean phase angle of 332 ± 80° (*n* = 109,559). After ripple-related (i.e., ± 250 ms) action potentials were removed the modulation of unit firing remained, but the magnitude was lower (*Z* = 26.6, *p* < 1e-9) and the mean phase angle was similar (357 ± 80°; *n* = 76,158). For the 20 neurons in the NSOZ, unit firing was also modulated by the slow wave, but the magnitude was much lower than in the SOZ (*Z* = 12.6, *p* < 0.001) and the highest firing probability shifted to 25 ± 80° (*n* = 38,019). After removing ripple-related action potentials, unit firing modulation decreased (*Z* = 9.74, *p* < 0.001) but the mean phase angle was similar (50 ± 80°; *n* = 28,080).

**Figure 3:**
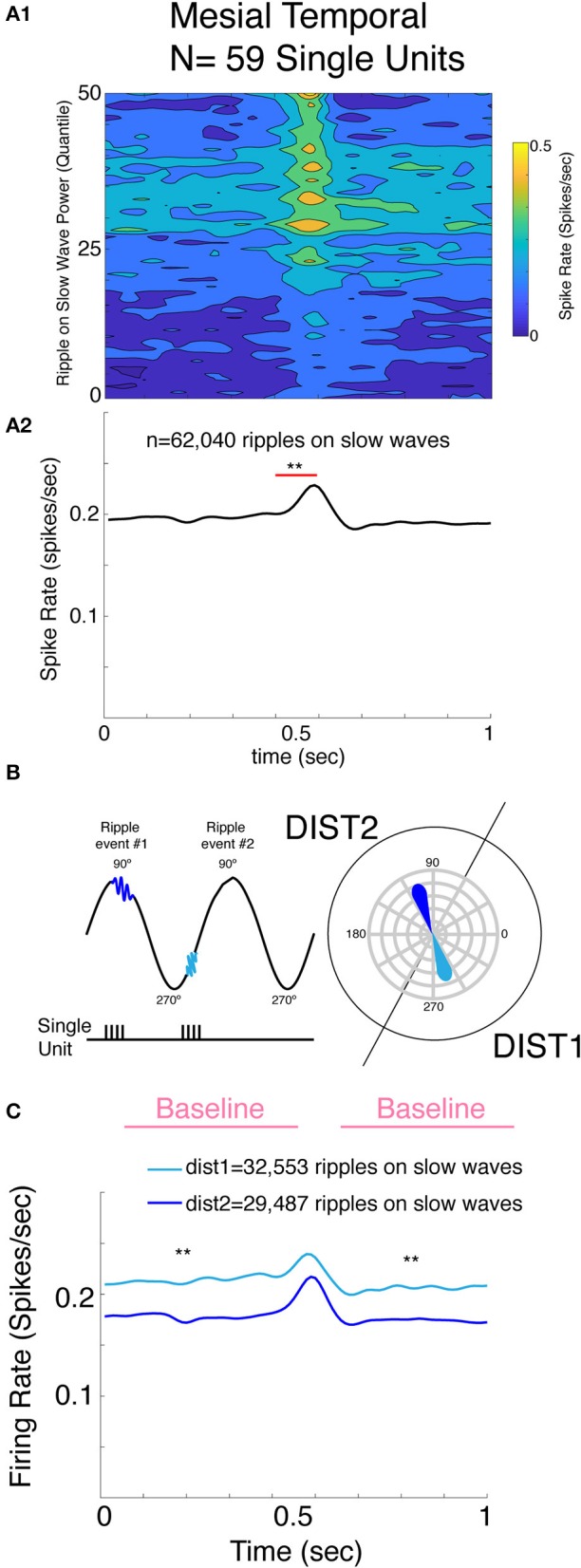
Mesial temporal lobe single unit spiking increases proportional to the RoSW power recorded from the adjacent macroelectrode, and units are only weakly modulated by the On and Off state. **(A)** Mesial temporal single unit spike rates increased around RoSW onset (*p* < 0.001, **A1,A2**). The increase in firing was proportional to the power of the RoSW recorded by the macroelectrode (*p* < 0.001, **A1**). **(B)** Illustration of derivation of single unit firing on the unit circle and definition of distribution 1 (DIST1) and DIST2. Note the two RoSW events and corresponding phasors on the unit circle. **(C)** Baseline mesial temporal single unit spike rate was greater for RoSW in DIST1 (cyan) than RoSW in DIST2 (blue, *p* < 0.001). However, the increase in the firing rate during the ripple with respect to the baseline firing was not statistically different. **p < 0.001.

Next, we examined the firing rate from all of the excitatory single units during RoSW using a linear mixed-effects model. The lone inhibitory unit precluded any meaningful analysis of this cell type. We found that all 59 excitatory single units firing increased at the time of the RoSW (*n* = 62,040 RoSW, *p* < 0.001, Figure 3A). Moreover, the increase in the excitatory neuron firing rate correlated with greater iEEG RoSW power (*F* = 41.26, *p* < 0.001, Figure 3A1) and was dependent on unit identity (i.e., unit number, *p* < 0.005). Neither the location of the unit nor the SOZ had an effect on excitatory firing, demonstrating that individual single units had diverse firing properties during the local RoSW. Thirteen out of fifty-nine of the units (22%) consistently fired during each RoSW recorded by the macroelectrode.

Similar to hippocampal RoSW in the larger cohort of patients, ripples occurred during all phase angles of the slow wave irrespective of the neuroanatomic location of the macroelectrode. Thus, for the next analysis, we separated RoSW in to two distributions, labeled Dist1 and Dist2, based on the phase-amplitude coupling results illustrated in Figure 1C3. Dist1 consisted of RoSW during the trough-peak (250–70°) transition and Dist2 were RoSW during the peak-trough (70–250°) transition (Figure 3B). The axis was shifted slightly to reflect the deviation evident in the data. Analysis found an increase in spike firing during RoSW with respect to baseline that was similar for Dist1 and Dist2 (*p* = 0.11, Figure 3C). Neither the neuroanatomical location of the single unit nor the location of the SOZ had an effect on excitatory unit firing during the RoSW (*p* > 0.05).

Lastly, the firing rate of the excitatory single units preceding and following RoSW (±500 ms) was significantly greater in Dist1 than in Dist2 (*p* < 0.001, Figure 3C), as expected based on the robust change in firing rates associated with different phases of slow wave activity (19). The increased baseline firing rate of excitatory single units for RoSW in Dist1 was not significantly correlated with the neuroanatomical location of the excitatory single unit (*p* > 0.05) or the location of the SOZ (*p* > 0.05).

## Discussion

We show in the hippocampal SOZ and NSOZ, fast ripples occur preferentially during the trough-peak or On-Off state transition of the slow wave. In the SOZ, RoSW also have a higher probability of coupling during the On-Off state, but in the NSOZ, RoSW are more likely to couple during the Off-On state transition. The wide range of phase angles associated with RoSW could be due to a mixture of pathological and normal ripples in epileptogenic and irritative tissue (1–3). Hippocampal RoSW during the On-Off state transition found here is consistent with results of neocortical RoSW in prior studies that show that ripples in the SOZ or resected regions are more likely to be coupled to the On-Off state, whereas ripples in healthy brain regions are more likely to be coupled to the Off-On state (4–6). These results may signify that the On-Off transition provides a more powerful depolarizing volley that promotes their generation (19).

The mechanisms responsible for ripple and fast ripple coupling with the slow wave were not fully elucidated in this study. The increase in excitatory single unit firing was similar for RoSW during the Off-On and the On-Off transition, but ripples during the On-Off transition had higher background firing rates. It is unlikely ripples alone could explain differences in background firing since removal of ripple-related firing only reduced, but did not eliminate, firing modulation. Rather, unit firing is also modulated by the slow wave and the fact that modulation of excitatory unit firing was stronger in the SOZ could be one factor contributing to the generation of pathological ripples.

Overall, only a minority of recorded neurons participated in ripple generation as reflected by the weak, yet significant, modulation (24). Interestingly, recent work in epileptic rats found pathological ripples recruit fewer neurons than ripples in healthy rats (25). Thus, in patients with epilepsy, pathological ripples might also recruit fewer neurons than normal ripples. This concept is inconsistent with a prior report (26) and what would be expected during fast ripples (15). However, in support of this concept, we found hippocampal ripples in the SOZ had lower spectral power and higher spectral frequency than ripples in the NSOZ, and spectral power was proportional to the increase in excitatory neuron firing (27). Fast ripples and unit firing were not studied here due to the challenges of isolating single unit waveforms during the fast ripple field potential, which represents population spikes consisting of summated neuronal spikes (15). Our results of RoSW in the NSOZ recapitulate other studies of normal hippocampal ripples on sharp waves that occur preferentially during the Off-On state transition (13). We also found longer duration ripples in the NSOZ than in the SOZ, which is consistent with the results from others (28).

In clinical epilepsy, the hippocampus may not be the ideal location to utilize slow wave phase-amplitude coupling to distinguish normal from pathological ripples (5). One reason may be that local slow waves propagate throughout the mesial temporal lobe and only moderately influence baseline firing rate (19). Another could be the architecture of the hippocampus and non-orthogonal orientation of the electrodes in relation to the cell layers and dipole generators. This could increase variability between patients and the slow wave On-Off state transition. Despite these technical issues, quantifying phase-amplitude coupling between slow waves and fast ripples has been shown to correlate with severity of epileptogenicity in patients with epileptic spasms (29), and our study suggests that these measures could also assist in surgical planning for mesial-temporal lobe epilepsy. RoSW phase-amplitude coupling may also assist researchers in identifying physiological ripples associated with memory encoding, consolidation, and recall in the human hippocampus. Our results are similar to those from human memory studies and suggest phase-amplitude could provide additional information for identifying physiological ripples in the human hippocampus (30).

## Data Availability Statement

The raw data supporting the conclusions of this article will be made available by the authors, without undue reservation, to any qualified researcher.

## Ethics Statement

The studies involving human participants were reviewed and approved by UCLA and TJU. The patients/participants provided their written informed consent to participate in this study.

## Author Contributions

SW, IS, and RS conceived and designed the experiments. AS, CW, and IF performed the experiments, the remainder of the authors analyzed the data. SW and RS wrote the manuscript.

### Conflict of Interest

SW is founder of Fastwave LLC, a Neurology software company. ZW and DS are cofounders and hold more than 5% equity interest in Fastwave LLC. The remaining authors declare that the research was conducted in the absence of any commercial or financial relationships that could be construed as a potential conflict of interest.
