# Supplementary material for: Ripples Have Distinct Spectral Properties and Phase-Amplitude Coupling With Slow Waves, but Indistinct Unit Firing, in Human Epileptogenic Hippocampus
Source: Front Neurol. 2020 Mar 24;11:174. doi: 10.3389/fneur.2020.00174 (PMC7118726; doi:10.3389/fneur.2020.00174)
Supplement: Supplementary file 1 [file Table_1.DOCX]

**Supplementary Methods**

**Supplementary Results**

| Patient Age/Sex Race | Risk Fac- tor | MRI | PET  Hypomet- abolic | iEEG IEDs | iEEG SOZs | Surgery | Pathology | Outcome |
| --- | --- | --- | --- | --- | --- | --- | --- | --- |
| 1. 3444   49/M W | None | cavernoma | left temporal | left MT left insular | left insula | left frontal resection | cavernous hemangioma | Engel I@24 |
| 1. 3759   45/M AA | None | Normal | right temporal | right MT frontal | right MT, insular | right ATL, insular resec- tion | gliosis | N/A |
| 1. 3967   27/M W | None | prior ant. 2/3 corpus callo- sotomy | N/A | right MT, left MT, insula | right occipital, left temporal, | None | N/A | N/A |
| 1. 3997   54/F W | febrile sei- zures | encephaloma- lacia/gliosis | N/A | left MT, right tempo- ral, insula | right insula | right ATL, insular resec- tion | gliosis | Engel III@36 |
| 1. 4036   34/M W | None | right hip- pocampal atrophy | right temporal | left and right temporal | left and right temporal | none | N/A | N/A |
| 1. 4050   25/M W | None | encephaloma- lacia/gliosis | normal | left MT, cin- gulate,parietal | left cingulate, parietal | VNS | N/A | N/A |
| 1. 4085   23/F W | infarction | left MTS, encephaloma- lacia | normal | left>right MT | left MT | left ATL | gliosis | Engel I@18 |
| 1. 4099   25/M W | None | Normal | right temporal | left MT | left MT | left ATL | gliosis | Engel I@27 |
| 1. 4110   42/F A | encephalitis | encephaloma- lacia/gliosis | normal | left MT, insular | left MT, insula | none | N/A | N/A |
| 1. 4122   19/M W | None | normal | right temporal | right tempo- ral, parietal | right posteri- or temporal, parietal | modified right temporal lobectomy | gliosis | Engel II@18 |

| Patient Age/Sex Race | Risk Fac- tor | MRI | PET  Hypomet- abolic | iEEG IEDs | iEEG SOZs | Surgery | Pathology | Outcome |
| --- | --- | --- | --- | --- | --- | --- | --- | --- |
| 1. 4163   58/F  W | SAH | encephaloma- lacia/gliosis | left temporal | left MT, frontal | left frontal | left frontal lobe resection, RNS | gliosis | Engel I@18 |
| 1. 4166   49/F  H | meningitis | encephaloma- cia/gliosis | left temporal | left MT, frontal | left MT, frontal | left MT, frontal | gliosis | Engel III@18 |
| 1. 448   58/M W | infarction | left MTS, encephaloma- lacia/gliosis | left temporal | left temporal | left MT | left ATL | gliosis | Death@10 |
| 1. 449   32/M H | None | encephaloma- lacia gliosis | left temporal, parietal | left insular parietal | left insular parietal | left parietal, RNS | gliosis | Engel I@26 |
| 1. 451   55/M W | perinatal distress | normal | left temporal | bilateral MT | bilateral MT | none | N/A | N/A |
| 1. 453   44/M A | None | abnormal T2 signal | right temporal T2 | bilateral MT | right MT | right ATL | cortical dys- plasia | Engel 1@28 |
| 1. 454   48/M W | None | L MTS, and extra-tempo- ral T2 | left temporal, frontal | left MT, insu- lar, orbitof- rontal | left MT and insular | RNS | N/A | N/A |
| 1. 456   37/F A | None | normal | right temporal | bilateral MT | bilateral MT R>L | modified R ATL | gliosis | Engel III@6 |
| 1. 458   33/M W | None | normal | right temporal | bilateral MT | bilateral tem- poral | RNS | N/A | N/A |
| 1. 463   27/M W | AVM | right occipital AVM | right occipital | bilateral MT | bilateral MT | RNS | N/A | N/A |
| 1. 466   21/F W | None | normal | left temporal | right MT, orbitofron- tal, frontal, cingulate | right temporal | right ATL | MTS | Engel I@23 |
| 1. 467   21/F W | None | left extra-tem- poral T2 | left parietal | left parietal | left parietal | left parietal, RNS | coritcal dys- plasia | Engel III@18 |
| 1. 468   50/M W | None | left MT FLAIR | right and left temporal | bilateral MT | bilateral MT | RNS | N/A | N/A |
| 1. 470   51/F W | None | left MTS | left temporal | left MT | left MT | RNS | N/A | N/A |
| 1. 473   70/F W | TBI w/ LOC | left MTS, extra-tempo- ral T2 | left temporal and frontal | bilateral MT | left MT | left visualase | N/A | Engel III@18 |

| Patient Age/Sex Race | Risk Fac- tor | MRI | PET  Hy- pometa- bolic | iEEG IEDs | iEEG SOZs | Surgery | Pathology | Outcome |
| --- | --- | --- | --- | --- | --- | --- | --- | --- |
| 1. 474   40/F W | TBI w/o LOC | vascular mal- formation | right frontal, temporal, parietal | bilateral temporal | bilateral temporal | none | N/A | N/A |
| 1. 477   45/F AA | None | periventric- ular nodular heterotopia, righ frontal T2 | right tem- poral | right MT right frontal | right MT | right ATL | normal | Engel III@11 |
| 1. 478   33/F W | None | periventric- ular nodular heterotopia, hypothalamic heterotopia | normal | left temporal, left frontal | left temporal, left frontal | RNS | N/A | N/A |
| 1. 479   36/M H | TBI w/ LOC | encepha- lomalacia/ gliosis | right tem- poral | right and left temporal neocortical | right tempo- ral neocor- tical | modified R ATL | gliosis | Engel II@10 |
| 1. 480   29/M W | perinatal distress | right hemi- sphere atro- phy, periven- tricular microgyria | right hemi- sphere | right occipital  , parietal, temporal | right tempo- ral neocor- tical | R ATL | gliosis | Engel III@10 |
| 1. 481   29/M W | TBI w/o LOC | left MTS | right and left temporal | left temporal | left temporal | RNS | N/A | N/A |
| 32.  IO001 52/F W | minor TBI | Normal | left temporal | left and right MT | left MT | modified L ATL | gliosis | Engel I@18 |
| 33.  IO002 52/M W | TBI w/ LOC | right hip- pocampal atrophy | right tem- poral | left and right MT | left and right MT | R ATL | gliosis | Engel I@18 |
| 34  IO004 29/M W | None | Normal | left temporal and occipital | left MT occipital/pa- rietal | left occipital | L posterior temporal, and occipital | cortical dys- plasia | Engel I@14 |
| 35  IO005 42/M W | febrile sei- zures | prior hippo- campal spar- ing temporal lobectomy | N/A | right MT | right MT | R ATL | gliosis | Engel III@14 |
| 36  IO009 20/M W | tuberous sclerosis | focal cortical dysplasia, nodules, giant cell tumors | left temporal | left temporal, parietal, right frontal | left temporal neocortical | none | N/A | N/A |
| 37  IO010 23/M W | septo-optic dysplastia | right periven- tricular pachygyria, deficiency  of septum pellucidum | N/A | right MT | right frontal | right frontal, RNS | gliosis | Engel III@12 |

Supplementary Table 1: **Patient characteristics of the iEEG sleep recording cohort.** Abbreviations (W: White, AA: African-American, A: Asian, H: Hispanic, R: right, L: left, TBI: traumatic-brain injury, LOC: loss of consciousness, SAH: sub-arachnoid hemorrhage, AVM: atriovenous hemorrhage, MTS: mesial-temporal lobe sclerosis, ATL: anterior temporal lobectomy, MT: mesial-temporal, VNS: vagal nerve stimulator, RNS: responsive nerve stimulator, N/A not applicable).

| Patient Age  Sex | MRI/PET | iEEG Seizure Onset | Resection Outcome | Recording Details | # Slow wave - Ripples |
| --- | --- | --- | --- | --- | --- |
| 398  41/F | Metabolic abnor- malities bilateral temporal lobe, nor- mal structural | Left temporal | Left temporal/Sig- nificant improvement | excitatory s.u.: 11  multiunit: 9  20 minutes  non-REM sleep | 4216 |
| 406  33/F | Mild metabolic abnormality left temporal, structural | Right mesial tem- poral | No surgery | excitatory s.u: 26  inhibitory s.u: 1  multiunit: 11 | 8295 |
|  | abnormality right |  |  | 50 minutes |  |
|  | frontal lobe |  |  | non-REM sleep |  |
| 417  23/M | Metabolic abnor- mality right parietal lobe, normal struc- | Right parietal | Right parietal, sei- zure free | excitatory s.u: 3  multiunit: 7  150 minutes non- | 1034 |
|  | Tural |  |  | REM sleep |  |
| 422  19/F | Normal | Bilateral multifocal | No surgery | excitaory s.u: 8  multiunit: 10  100 minutes non- | 27,188 |
|  |  |  |  | REM sleep |  |
| 423  52/F | Metabolic and structural abnormalities left temporal lobe | Left mesial-temporal | Left temporal/Sei- zure free | excitatory s.u: 11  multiunit: 15  110 minutes non- REM sleep | 21,307 |

Supplementary Table 2: **Patient characteristics of the iEEG-LFP sleep recording cohort.** Abbreviations (s.u: single unit).
